# Selective Copper Removal from an Fe–P–Cu Alloy Recovered by Pyrometallurgical Reduction of Spent LiFePO_4_ Batteries via Sulfidation–Slag Refining

**DOI:** 10.3390/ma19061185

**Published:** 2026-03-18

**Authors:** Jin-Seong Yoon, A-Jin Im, Jei-Pil Wang

**Affiliations:** Department of Metallurgical Engineering, BB21 Plus Team, Pukyong National University, Busan 48513, Republic of Korea

**Keywords:** spent LiFePO_4_ (LFP) battery, pyrometallurgy, sulfidation–slag refining, Fe–P–Cu alloy, fayalite slag, selective Cu removal

## Abstract

The recycling of spent lithium iron phosphate (LiFePO_4_, LFP) batteries is receiving increasing attention as electric-vehicle deployment accelerates worldwide. Pyrometallurgical reduction offers a viable route for large-scale recovery of iron-rich products from spent LFP batteries; however, the resulting Fe-based alloys often retain residual copper (Cu), which deteriorates alloy quality and constrains downstream utilization and refining. In this study, a sulfidation–slag refining process was developed to selectively remove Cu from an Fe–P–Cu alloy produced by dry reduction of spent LFP batteries. FeS was employed as a sulfidizing agent to promote preferential conversion of Cu into sulfide phases, while fayalite (Fe_2_SiO_4_) slag was introduced to enhance phase separation between metallic and sulfide/slag phases. Thermodynamic calculations coupled with high-temperature experiments were conducted at 1400–1600 °C under various Cu:FeS ratios to identify operating conditions that maximize Cu removal while minimizing Fe loss. The results indicate that Cu is selectively transferred from the metallic phase to Cu–Fe–S sulfide phases, whereas Fe remains predominantly in the metal phase. Under the optimal condition (1400 °C, Cu:FeS = 2:1), the refined metal reached an Fe content of 90.80 wt.%, achieving an Fe recovery of 87.42% and a Cu removal efficiency of 81.13%. The proposed approach provides a practical stepwise refining strategy for upgrading Fe-rich secondary resources recovered from spent LFP batteries and facilitates subsequent impurity-control processes.

## 1. Introduction

The rapid global deployment of electric vehicles (EVs) is driving a sharp increase in lithium-ion battery (LIB) production and, consequently, the future generation of large volumes of end-of-life batteries [1,2,3]. Anticipated surges in spent LIB streams after 2030 have intensified the need for recycling technologies that simultaneously mitigate environmental risks and secure secondary resources [4,5]. Beyond preventing the release of hazardous constituents during disposal, battery recycling is increasingly regarded as a key enabler of a circular economy through the recovery and reintegration of critical and bulk materials [6,7,8]. Recent industrial-scale and life-cycle assessments further indicate that recycling route selection can substantially influence environmental impacts, reinforcing the need for robust and scalable recycling technologies [9].

Among commercial LIB chemistries, lithium iron phosphate LiFePO_4_ (LFP) batteries are gaining market share, particularly for EVs and stationary energy storage systems, owing to their thermal stability, long cycle life, and reduced reliance on expensive Ni- and Co-bearing cathodes [10,11,12,13]. However, the absence of high-value metals also undermines the economics of conventional hydrometallurgical recycling routes that were primarily developed for Ni/Co-rich cathodes [14,15]. Although numerous studies have reported aqueous leaching-based recovery of Li, Fe, and P from spent LFP as well as direct regeneration of LFP cathode materials, these approaches may suffer from high reagent consumption, wastewater generation, process complexity, or performance degradation and impurity accumulation during repeated regeneration cycles [16]. Therefore, alternative recycling strategies that create value from the Fe-rich nature of LFP materials are required [17].

Pyrometallurgical reduction has recently attracted attention as a robust route for treating spent LFP batteries because it enables large-scale processing with simplified flowsheets and minimal wastewater generation [18,19,20]. In this approach, the cathode-derived iron can be recovered directly as an Fe-based alloy. Nevertheless, the reduced product commonly contains impurity elements such as copper (Cu) and phosphorus (P), forming an Fe–P–Cu alloy rather than a clean Fe resource [21,22]. Residual Cu is particularly detrimental because it degrades the quality and downstream usability of Fe-based metals and can complicate subsequent refining steps aimed at controlling P. Accordingly, the practical utilization of Fe recovered from spent LFP batteries requires a stepwise refining strategy in which Cu is selectively removed prior to further impurity management.

Increasing attention has also been paid to the management and valorization of FePO_4_-rich residues generated after Li recovery from LFP streams, underscoring the importance of Fe-centered pathways and impurity control [23,24]. Selective sulfidation is a promising option for Cu removal because Cu generally exhibits a higher affinity for sulfur than Fe, allowing Cu to be preferentially converted to sulfide species under appropriate conditions [25,26,27]. Such sulfidation-based refining principles have long been exploited in metallurgical operations to partition Cu into a sulfide phase separable from the metallic phase. In addition, stable phase separation during sulfidation can be enhanced by controlling the slag system. In particular, fayalite (Fe_2_SiO_4_)-based slags—commonly generated as by-products in copper smelting—offer an FeO–SiO_2_ matrix that can facilitate liquid-phase formation and promote separation between metal and sulfide/oxide melts at high temperatures.

In this study, a sulfidation–slag refining process using FeS as a sulfidizing agent and fayalite slag as a separation-promoting medium was developed to selectively remove Cu from an Fe–P–Cu alloy recovered from spent LFP batteries via a pyrometallurgical (non-aqueous) route. The work provides a scientifically grounded basis for stepwise upgrading of Fe-rich secondary resources and for subsequent impurity-control strategies.

## 2. Materials and Methods

### 2.1. Preparation of the Fe–P–Cu Alloy from Spent LFP Batteries

**Figure 1:**
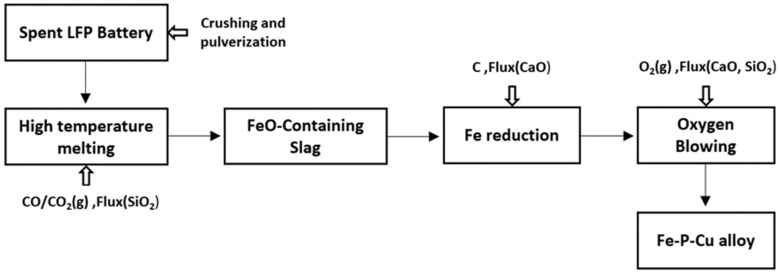
Flowchart of the stepwise pyrometallurgical route used to prepare the Fe–P–Cu alloy from spent LFP batteries.

An Fe–P–Cu alloy was prepared from spent lithium iron phosphate (LFP) batteries via a stepwise pyrometallurgical route consisting of (i) battery crushing, (ii) high-temperature melting to form an FeO-containing slag, (iii) reduction of the slag to produce Fe-based molten metal, and (iv) oxygen blowing (O_2_ blowing) to stabilize the metal composition and separate phosphorus. A schematic of the overall procedure is provided in Figure 1.

**Figure 2:**
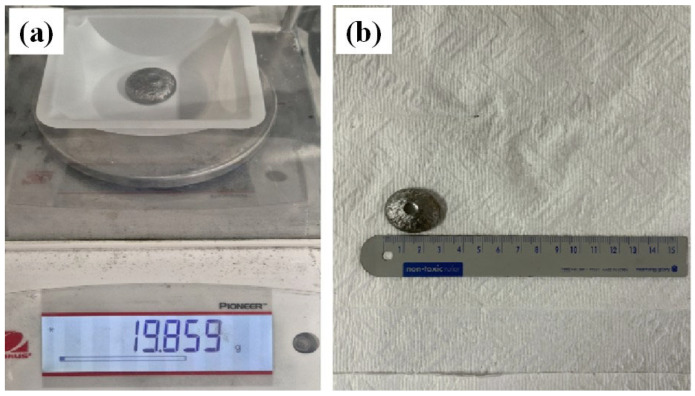
Photographs of the Fe–P–Cu reference alloy produced from spent LFP batteries via a stepwise pyrometallurgical (non-aqueous) route (reduction followed by oxygen blowing): (**a**) mass measurement of the recovered alloy; (**b**) representative alloy button (scale shown).

Prior to crushing, residual voltage in the spent LFP batteries was fully removed by electrical discharge to 0 V to ensure safe handling. Crushing was conducted in batches of 10 kg. Five intact battery cells (approximately 2 kg in total) were charged without disassembly and comminuted by repeated forward/reverse rotation; complete crushing was achieved within ~3 min. The recovered crushed product was used as the feed for subsequent high-temperature melting. Crushing was conducted in batch mode (10 kg) to reflect practical preprocessing of intact cells and to ensure safe handling after complete discharge. Subsequent high-temperature steps were performed on laboratory-scale sub-samples taken from the crushed product under controlled conditions; Figure 2 shows a representative recovered alloy button used for baseline composition determination and experimental design. Detailed bulk compositional analysis of the entire crushed feed was not included in the present scope; instead, the composition of the recovered alloy after oxygen blowing (Table 1) was used as the reference basis for subsequent controlled refining experiments. The crushed product was converted into an oxide slag dominated by FeO through high-temperature melting under a controlled oxygen partial pressure using a CO/CO_2_ gas mixture. To prevent unintended reduction of iron during melting, the oxygen partial pressure was maintained at approximately 10^−11^ atm based on the Fe–FeO–Fe_3_O_4_ equilibrium. The slag composition was adjusted by adding SiO_2_ as a flux; after carbon removal, 11.05 g of SiO_2_ was added per 52 g of the crushed product. Here, “carbon removal” denotes the oxidation/removal of carbonaceous species contained in the crushed battery feed, primarily anode-derived graphite and conductive/binder-related carbon (e.g., carbon black and binder residues), to avoid unintended carbothermic reduction during the controlled oxygen-potential melting stage. The furnace was heated at a ramp rate of 10 °C min^−1^ from room temperature to 1350 °C, followed by a 30 min stabilization period to ensure thermal equilibration and stable gas flow before applying the controlled CO/CO_2_ atmosphere for oxygen potential regulation. The melting experiment was then performed at 1350 °C for 10 h. The chemical composition of the resulting FeO-containing slag was determined by X-ray fluorescence (XRF), showing FeO 43.10 wt.%, P_2_O_5_ 26.70 wt.%, SiO_2_ 23.56 wt.%, and CuO 4.61 wt.% as the major components. The FeO-containing slag was then reduced to produce an Fe-based molten metal. For the reduction experiment, 47.285 g of slag was mixed with 7.0 g of carbon as a reductant and 22.715 g of CaO as a flux, and the slag basicity (CaO/SiO_2_) was adjusted to 2.0. This corresponds to 14.8 wt.% C and 48.0 wt.% CaO relative to the slag charge (mass basis). The reductant dosage was defined by the intentionally added carbon; the carbon crucible served primarily as a container, and the short holding time (30 min) was employed to minimize uncontrolled additional carbon contribution. The carbon addition was selected based on the stoichiometric requirement for FeO reduction (FeO + C → Fe + CO) using the measured FeO content of the slag, with an excess factor to ensure sufficient reduction under the present experimental conditions. The mixture was charged into a carbon crucible and heated to 1600 °C in a high-frequency induction furnace under an Ar atmosphere (300 cc min^−1^), and held for 30 min. The reduced Fe-based molten metal was subsequently subjected to oxygen blowing to stabilize its composition and promote phosphorus removal. Oxygen blowing was conducted using 30 g of the Fe–P-based metal with the addition of 6.6 g CaO and 3.3 g SiO_2_ as fluxes. At 1600 °C, O_2_ gas was injected at 300 cm^3^ min^−1^ for 10 min, followed by a holding period of 20 min at the same temperature to ensure sufficient metal–slag reaction. During oxygen blowing, the lance tip was positioned 10 mm above the melt surface (corresponding to an insertion depth of 50 mm from the furnace top reference), and the O_2_ injection angle was set to 0° (vertical downward) relative to the vertical axis. During oxygen blowing, phosphorus in the metal was selectively oxidized and transferred to the slag phase. The recovered metal after oxygen blowing consisted primarily of Fe and contained 7.57 wt.% P and 6.44 wt.% Cu (Figure 2). The Fe–P–Cu alloy obtained after oxygen blowing (Figure 2) was used as the reference alloy to define the target bulk composition (Table 1) for the subsequent sulfidation–slag refining study. This reference composition was then used to design the starting metallic charge for the subsequent sulfidation–slag refining experiments.

### 2.2. Experimental Materials

Figure 2 shows the Fe–P–Cu alloy obtained via the dry reduction route applied to spent LFP batteries. This alloy was used as the reference alloy in the present work and served as the baseline composition for designing subsequent sulfidation–slag refining experiments.

The chemical composition of the reference alloy was quantified by inductively coupled plasma–optical emission spectroscopy (ICP–OES), and the results are summarized in Table 1. The reference alloy consisted primarily of Fe (85.89 wt.%), with P and Cu contents of 7.57 wt.% and 6.44 wt.%, respectively.

To clearly observe phase-separation behavior among the metallic phase, the sulfide (matte) phase containing Cu_2_S, and the slag phase during sulfidation–slag refining, an alumina crucible (diameter: 3.2 cm; height: 4.7 cm) was employed. Considering melt stability and overflow prevention, the maximum practical charge was set to 250 g. Because only a limited amount of reference alloy could be recovered from the spent-battery reduction campaign and because repeated tests require identical starting composition and sufficient charge mass, composition-matched Fe–P–Cu alloys were synthesized for the refining experiments. These synthesized alloys reproduce the ICP–OES composition of the reference alloy (Table 1) and were used as the starting metallic charge for all sulfidation–slag tests unless otherwise stated. Accordingly, Fe–P–Cu alloys were synthesized to match the ICP–OES composition in Table 1 using Fe powder (99.4%, Duksan, Ansan, Republic of Korea), P powder (≥99%, Duksan, Ansan, Republic of Korea), and Cu powder (99.9%, Duksan, Ansan, Republic of Korea). The powders were weighed to the target composition, thoroughly blended, and used as the starting metallic charge. FeS powder (50 wt.% purity, Duksan, Ansan, Republic of Korea) was used as the sulfidizing agent. A fayalite-type slag was introduced to promote phase separation; the slag was supplied as an industrial byproduct from a domestic non-ferrous smelting process (Young Poong Corporation, Seokpo, Republic of Korea). X-ray diffraction (XRD) confirmed Fe_2_SiO_4_ as the major crystalline phase, and scanning electron microscopy with energy-dispersive spectroscopy (SEM–EDS) indicated a multicomponent Fe–Si–O-based slag. The detailed phase assemblage and chemical composition of the slag are discussed.

### 2.3. Experimental Apparatus

**Figure 3:**
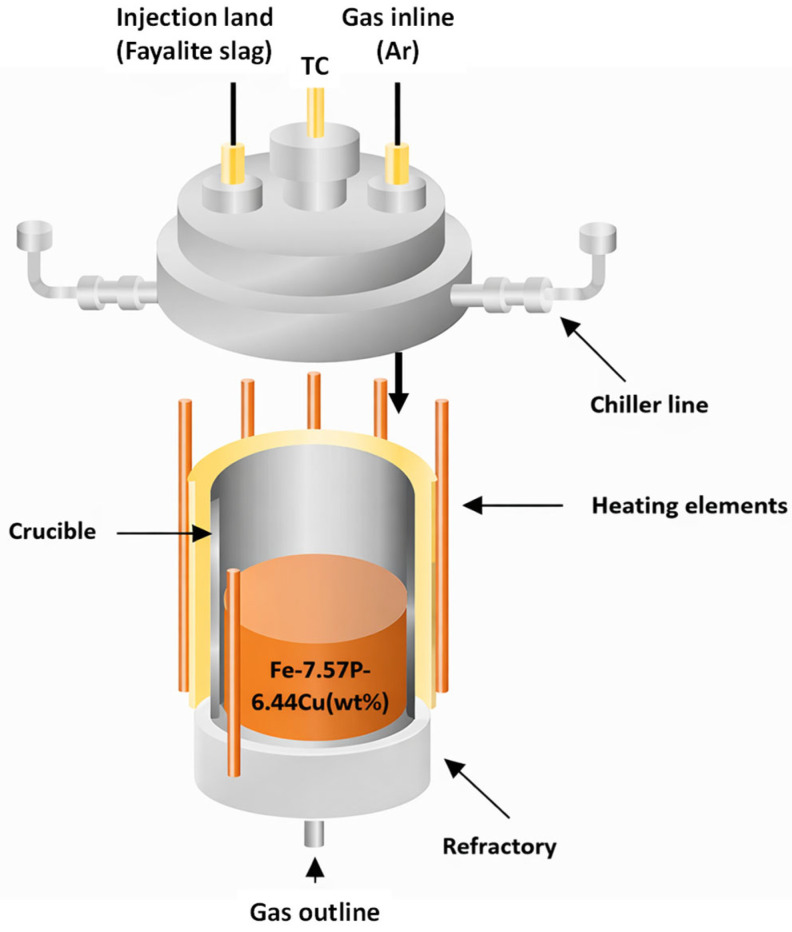
Schematic illustration of the Super Kanthal (MoSi_2_)-heated vertical tube furnace used for sulfidation–slag refining experiments.

Sulfidation–slag refining experiments were conducted in a vertical tube furnace equipped with Super Kanthal (MoSi_2_) heating elements, allowing stable temperature control up to 1600 °C. All experiments were performed under an inert Ar atmosphere. Argon was continuously introduced through the gas inlet at the top of the furnace and discharged through the outlet at the bottom, ensuring a continuous purge during heating and reaction. A schematic illustration of the experimental setup is provided in Figure 3.

The reaction charge was placed in an Al_2_O_3_ crucible positioned within a refractory support and located in the hot zone to ensure a uniform temperature field. For the slag-refining step following sulfidation, an injection lance was installed at the top of the furnace to deliver fayalite slag directly into the molten metal. The injection lance consisted of an Al_2_O_3_ (alumina) tube, selected for chemical compatibility with fayalite slag at high temperature. The Ar atmosphere was maintained throughout slag injection and the subsequent holding period to enable metal–sulfide–slag reactions and phase separation. Temperature was monitored and controlled in real time using a thermocouple inserted from the top of the furnace.

### 2.4. Thermodynamic Modeling

Thermodynamic calculations were conducted to (i) predict sulfidation reactions in the Fe–P–Cu alloy, (ii) evaluate phase partitioning and separation upon the addition of fayalite slag, and (iii) support the selection of experimental conditions. Calculations were performed using FactSage (FactSage 8.2, Thermfact/CRCT and GTT-Technologies, Montreal, QC, Canada). Equilibrium computations were carried out with the Equilib module by assuming multiphase equilibrium among the molten metal, sulfide-containing liquid phases, and oxide slag phases at the reaction temperature. The FactPS and FTmisc databases were used to describe metallic and sulfide species, while the FToxid database was employed for oxide slag phases. The principal phases considered included (i) a molten Fe–P–Cu metallic phase, (ii) a sulfide-rich liquid phase represented by Cu_2_S-containing liquids, and (iii) an oxide slag phase in the FeO–SiO_2_ system, with emphasis on fayalite slag stability and coexistence with sulfide/oxide liquids. Calculations were performed over 1400–1600 °C, corresponding to the experimental temperature range. The modeling results were used to estimate Cu transfer from the metal to sulfide phases via Cu_2_S formation and to anticipate phase separation among the metal, Cu_2_S–FeO-containing liquids, and fayalite slag after slag addition. Based on the calculations, three temperatures (1400, 1500, and 1600 °C) were selected for the primary experimental campaign.

### 2.5. Experimental Analysis

The chemical composition of the reference alloy was determined by ICP–OES (Optima 8300, PerkinElmer, Waltham, MA, USA), and the data were used for baseline characterization and for defining target compositions in subsequent experiments. Phase evolution before and after refining was examined by XRD (D8 Advance, Bruker, Karlsruhe, Germany) using Cu Kα radiation (λ = 1.5406 Å). Diffraction patterns were collected over 2θ = 10–80° with a step size of 0.02° and a scanning rate of 2° min^−1^, and crystalline phases were identified by comparison with the ICDD PDF database. Microstructural observations and local compositional analyses were performed by SEM–EDS using a tabletop SEM (EM-30N, COXEM, Daejeon, Republic of Korea) equipped with an EDS detector. SEM–EDS was used primarily for microstructural observation and semi-quantitative local composition assessment (elemental mapping and spot analyses). Because SEM–EDS can be limited for accurate bulk quantification of heterogeneous phases, bulk alloy composition was determined by ICP–OES, and SEM–EDS spot compositions were obtained from at least 10 points per phase (from at least three different areas) to improve representativeness and are reported as mean ± standard deviation where applicable. The recovered metal, solidified sulfide-derived phases, and slag were examined to assess phase distribution and local chemical partitioning.

### 2.6. Experimental Procedure

**Figure 4:**
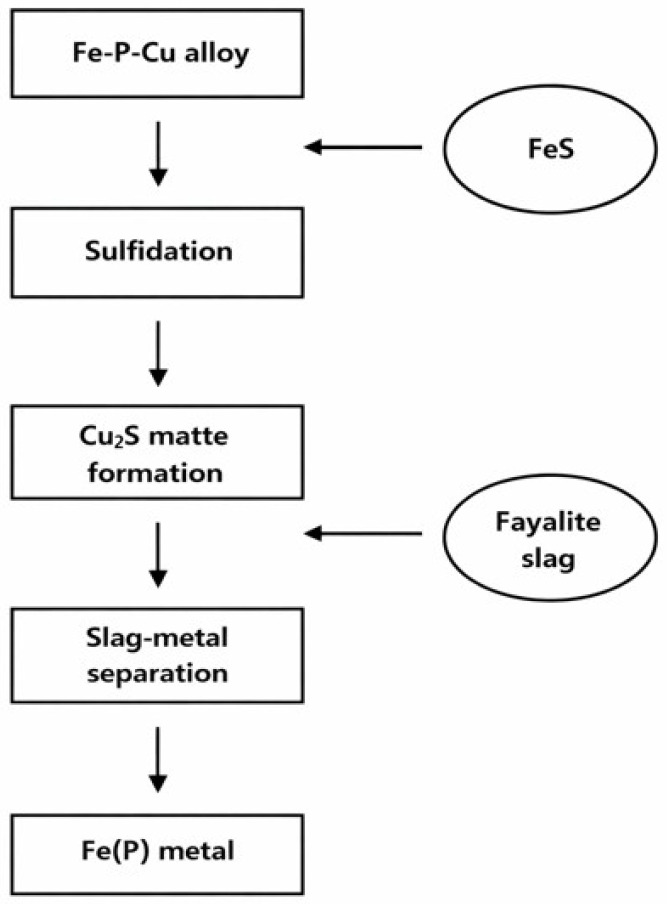
Schematic workflow of the sulfidation–slag refining process for selective Cu removal from the Fe–P–Cu alloy.

The overall experimental workflow for sulfidation–slag refining is schematically summarized in Figure 4. The refining experiments (sulfidation + slag refining) were conducted using the composition-matched Fe–P–Cu alloy described in Section 2.2, while the reference alloy produced from spent LFP batteries served to define the target composition and validate the relevance of the study to real feeds. The synthesized Fe–P–Cu alloy was charged into an Al_2_O_3_ crucible and placed in the vertical tube furnace. The furnace was purged with Ar to remove residual air, and Ar was continuously supplied at 300 cm^3^ min^−1^ throughout the experiment to maintain an inert atmosphere. The sample was heated to the target temperature. In the sulfidation step, FeS powder was added to the molten Fe–P–Cu alloy after reaching the set temperature. The reaction was maintained for 3 h at the same temperature, during which Cu in the metal preferentially reacted with sulfur to form Cu_2_S-containing sulfide phases. After sulfidation, the slag-refining step was initiated by adding dried solid fayalite slag through the injection lance (Al_2_O_3_ tube) installed at the top of the furnace, thereby delivering the slag charge into the hot zone and directly onto the molten metal. The system was held for an additional 3 h at the same temperature to promote reactions and phase separation among the molten metal, sulfide-rich liquids, and oxide slag. Upon completion, the sample was furnace-cooled under Ar. Cooling was conducted by uncontrolled furnace cooling; based on a separate blank run and furnace specifications, the average cooling rate was approximately 5 °C min^−1^ from 1600 to 1200 °C and 3 °C min^−1^ from 1200 to 800 °C. After cooling, the metallic, sulfide, and slag phases were physically separated. The final recovered metallic phase, containing residual phosphorus, is denoted as Fe(P) metal and was subjected to subsequent chemical and microstructural analyses. After physical separation, the sulfide-rich phase (Liquid#1) and the slag phase were collected separately and stored in sealed containers. In the laboratory, these by-products were handled and disposed of in accordance with institutional hazardous-waste management procedures. From a process perspective, the sulfide-rich phase can be regarded as a Cu-bearing matte and is potentially amenable to subsequent Cu recovery (e.g., oxidation/slagging or hydrometallurgical treatment depending on the facility), whereas the fayalite-based slag may be recycled to the slag system or stabilized and managed according to local regulations after verifying leaching stability. Each experimental condition was repeated n = 3 times. Reported values are presented as mean ± standard deviation. For compositional measurements, analytical uncertainty was assessed by ICP–OES replicate measurements and multiple-point SEM–EDS analyses per sample.

## 3. Results and Discussion

### 3.1. Characterization and Melting Behavior of Fayalite Slag

**Figure 5:**
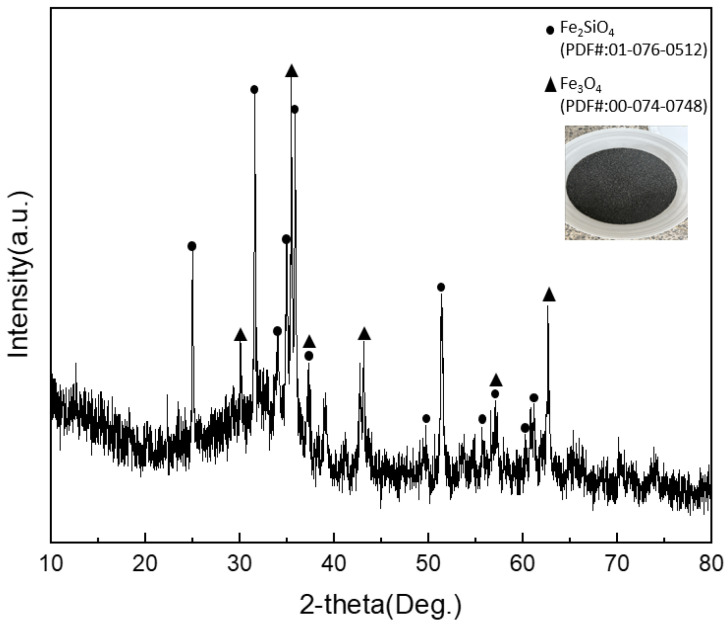
XRD pattern of the fayalite-based slag used in this study (major phase: Fe_2_SiO_4_; minor phase: Fe_3_O_4_).

To verify the phase constitution and chemical characteristics of the fayalite-based slag employed as the refining slag in this study, X-ray diffraction (XRD) and scanning electron microscopy coupled with energy-dispersive spectroscopy (SEM–EDS) were conducted. As shown in Figure 5, the diffraction peaks of the slag were predominantly indexed to fayalite (Fe_2_SiO_4_), indicating that the slag matrix is governed by a fayalite phase. In addition, weak reflections attributable to magnetite (Fe_3_O_4_) were detected as a secondary phase. The presence of magnetite is consistent with the partially oxidized nature commonly observed in industrial non-ferrous smelting slags and does not alter the identification of the slag as a fayalite-dominant system under the present conditions.

Local chemical composition of the representative phase was further examined by SEM–EDS, and the quantitative results are summarized in Table 2. After normalization to the Fe–Si–O system, the measured composition closely approached the stoichiometric ratio of Fe_2_SiO_4_, in agreement with the XRD results. Minor amounts of Al and C were also detected, which can be attributed to typical impurities originating from industrial slag feedstock and/or sample handling. Considering their low concentrations, these minor components were not expected to dominate the phase assemblage or significantly modify the melting behavior of the fayalite matrix within the temperature window investigated.

**Figure 6:**
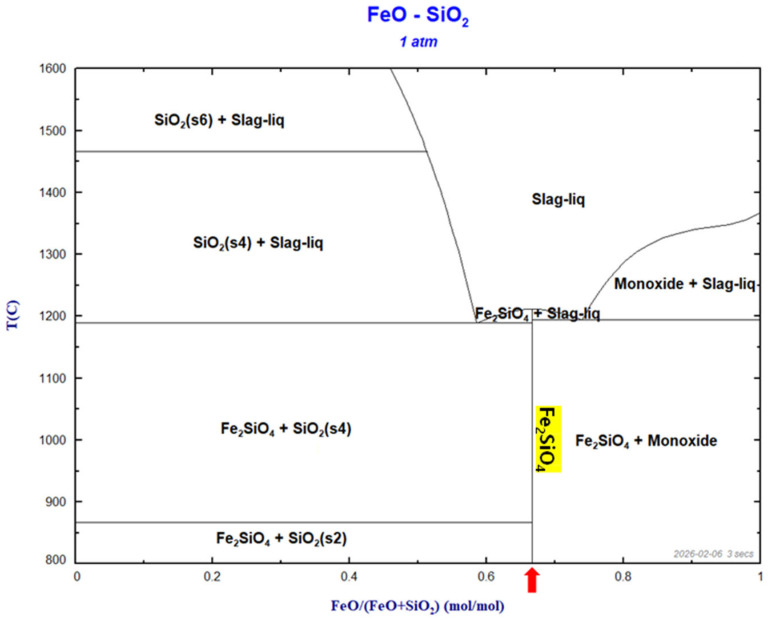
FeO–SiO_2_ binary phase diagram at 1 atm calculated using FactSage™, illustrating the melting behavior near the fayalite composition (Fe_2_SiO_4_). The red arrow indicates the Fe_2_SiO_4_ composition corresponding to the fayalite slag region.

The melting behavior of the slag was assessed based on the binary FeO–SiO_2_ phase diagram (Figure 6). In the FeO–SiO_2_ system, the composition near fayalite (Fe_2_SiO_4_) transitions to a liquid phase at approximately 1185–1205 °C, and a broad liquid field is retained above 1200 °C over a wide compositional range. This implies that, in the high-temperature regime adopted for the sulfidation–slag refining experiments, the fayalite-based slag can remain sufficiently molten, thereby providing adequate fluidity to promote interfacial reactions and phase separation. Therefore, from the standpoint of slag fusibility and stability, the fayalite slag employed herein is suitable for high-temperature refining where intimate contact between metal, sulfide, and slag phases is required. The detailed selection of the experimental temperature range is further discussed in the following section in conjunction with the thermodynamic feasibility of the sulfidation reactions.

### 3.2. Thermodynamic Feasibility of the Sulfidation Reaction

**Figure 7:**
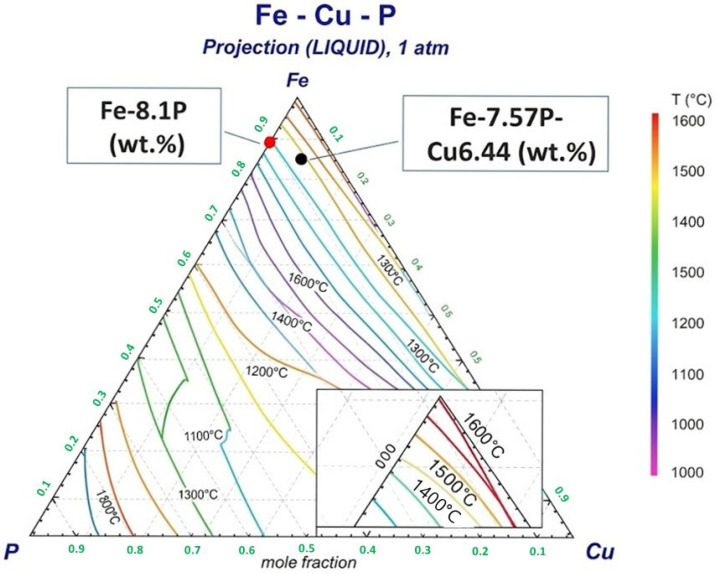
Liquidus projection of the Fe–Cu–P ternary system at 1 atm calculated using FactSage 8.2. The positions of the nominal alloy composition (Fe–7.57P–6.44Cu, wt.%) and the Cu-depleted Fe–P composition (Fe–8.1P, wt.%) are indicated.

Prior to applying the sulfidation–slag refining route, the melting stability of the Fe–P–Cu alloy and the thermodynamic feasibility of selective Cu sulfidation were evaluated. First, to confirm that the sulfidation proceeds in the liquid–metal state, liquidus calculations for the Fe–Cu–P ternary system were performed using FactSage 8.2. As shown in Figure 7, the nominal alloy composition employed in this study (Fe–7.57P–6.44Cu, wt.%) lies within the liquid phase field at temperatures above approximately 1400 °C, indicating that the alloy remains molten under the targeted reaction conditions. In addition, the Fe–P composition after Cu removal (i.e., Cu-depleted alloy) also stays in the liquid region over the same temperature range, implying that selective Cu removal by sulfidation is not expected to induce premature solidification of the metallic phase.

Next, the thermodynamic driving force for selective Cu removal was assessed by comparing the sulfidation tendencies of Fe and Cu, and by evaluating the exchange reaction for Cu sulfidation under FeS-containing conditions. The sulfidation reactions of Fe and Cu can be written as:

2Fe + S_2_(g) → 2FeS, (1)

4Cu + S_2_(g) → 2Cu_2_S, (2)

From these reactions, the preferential conversion of Cu to sulfide in the presence of FeS can be represented by the exchange reaction:

2Cu + FeS → Fe + Cu_2_S, (3)

**Figure 8:**
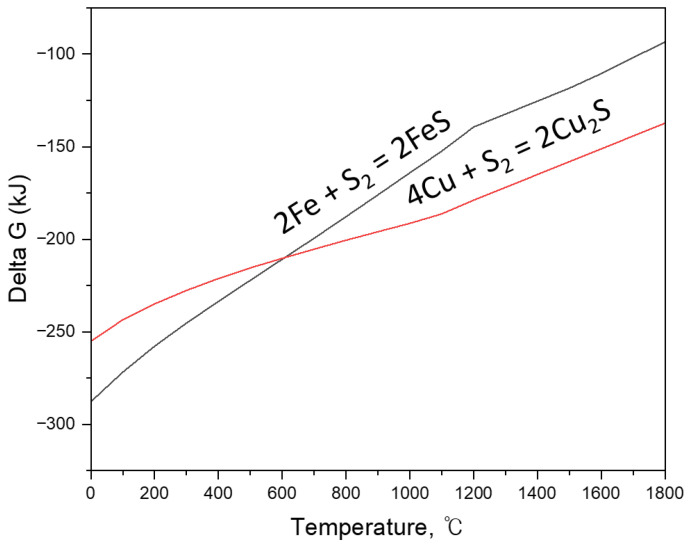
Standard Gibbs free energy changes (ΔG°) of the sulfidation reactions of Fe and Cu as a function of temperature.

Standard Gibbs free energy changes (ΔG°) for Equations (1)–(3) were calculated in the range of 1400–1600 °C, and the results are summarized in Table 3 and Figure 8. Table 3 provides the temperature-dependent ΔG° values for the individual sulfidation reactions of Fe and Cu and for the derived exchange reaction, while Figure 8 visualizes the relative thermodynamic driving forces as a function of temperature. The calculations show that Cu sulfidation (Equation (2)) exhibits more negative ΔG° values than Fe sulfidation (Equation (1)) across 1400–1600 °C, demonstrating a stronger affinity of Cu for sulfur compared to Fe under the examined conditions. More importantly, the exchange reaction (Equation (3)) remains thermodynamically spontaneous throughout the investigated temperature range, with ΔG° values of −19.781 kJ at 1400 °C, −19.816 kJ at 1500 °C, and −20.325 kJ at 1600 °C. These consistently negative values indicate that, once FeS is present, Cu can be preferentially converted to Cu2S while Fe is released back to the metallic phase. Based on the combined requirements of (i) maintaining a fully molten metallic phase and (ii) ensuring sufficient thermodynamic driving force for selective Cu sulfidation, three reaction temperatures (1400, 1500, and 1600 °C) were selected for the subsequent sulfidation–slag refining experiments.

### 3.3. Thermodynamic Analysis of Sulfidation and Slag–Liquid Equilibria

#### 3.3.1. Equilibrium Phase Distribution and Stabilization of the Cu_2_S-Bearing Liquid Phase

Equilibrium phase distributions for the Fe–P–Cu alloy in the presence of FeS and fayalite slag were calculated at 1400, 1500, and 1600 °C using FactSage 8.2. The phase amounts and equilibrium compositions of the sulfide-rich liquid (Liquid#1) and the slag liquid phase (Slag–liq#1) are summarized in Table 4. At all three temperatures, equilibrium was characterized by the coexistence of Liquid#1 and Slag–liq#1, indicating stable separation between a Cu–S–Fe–O-bearing liquid and an FeO–SiO_2_-based slag melt.

A compositional inspection of Liquid#1 reveals that the Cu-to-S molar ratio is close to ~2:1 across the investigated temperature range, while Fe and O appear in approximately ~1:1 molar proportion. These relationships suggest that, at the reaction temperature, Liquid#1 is not a purely sulfide melt but rather an oxide–sulfide mixed liquid in which Cu_2_S is stabilized in the presence of FeO (i.e., a Cu_2_S–FeO-bearing liquid). It should be noted that this description refers to the high-temperature liquid constitution predicted by thermodynamic equilibrium, whereas the crystalline phases observed at room temperature may differ due to redistribution and crystallization during furnace cooling (e.g., formation of Cu–Fe–S intermediate sulfides), as discussed later in the context of XRD results.

**Figure 9:**
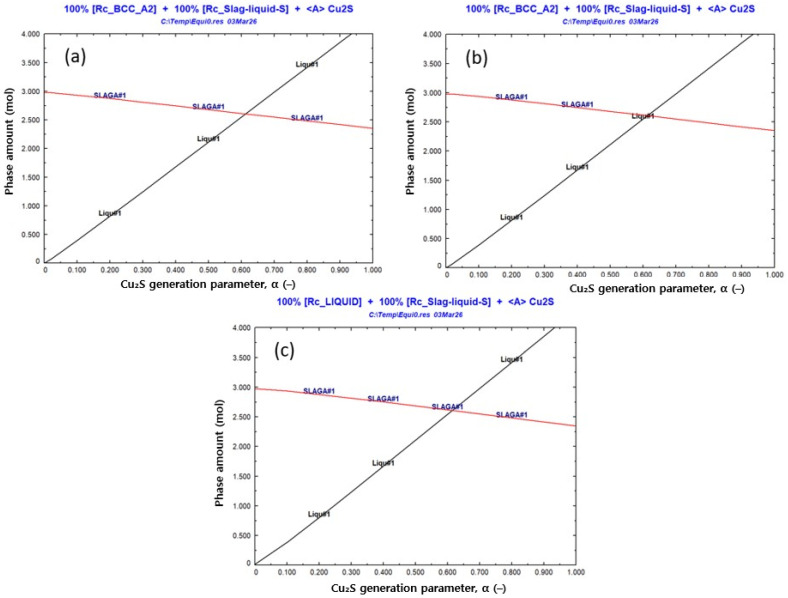
Effect of Cu_2_S generation parameter (α) on the phase amounts calculated by FactSage 8.2 at (**a**) 1400 °C, (**b**) 1500 °C, and (**c**) 1600 °C. Here, α is a normalized Cu_2_S-generation parameter (dimensionless, 0–1), and the phase amount is reported on a molar basis (mol).

To evaluate how Cu_2_S formation influences liquid-phase stabilization, additional equilibrium calculations were performed by treating the amount of generated Cu_2_S as a variable parameter, A (mol). Figure 9a–c present the variation in phase amounts with increasing A at 1400, 1500, and 1600 °C, respectively. At all temperatures, increasing A leads to a systematic increase in the amount of Liquid#1, demonstrating that higher Cu_2_S generation promotes the formation and stabilization of the oxide–sulfide mixed liquid. In contrast, Slag–liq#1 remains dominated by the FeO–SiO_2_ melt and shows comparatively moderate changes. These results identify Cu_2_S generation as a key factor governing the emergence and stability of Liquid#1 in the sulfidation–slag system, providing a thermodynamic basis for subsequent activity evaluation and slag-addition design.

#### 3.3.2. Activity and Gibbs Free Energy Analysis of the Liquid Phase

The activities of Cu, Fe, O, and S in Liquid#1 were evaluated at 1400–1600 °C to quantify the thermodynamic stabilization of the molten phase. As summarized in Table 5, all activities were substantially lower than unity, indicating strong non-ideal interactions in the melt. In particular, the relatively low activities of Cu and S suggest pronounced Cu–S association in Liquid#1, consistent with sulfide-like short-range ordering. Meanwhile, the extremely low activities of Fe and O reflect strong Fe–O interactions under the prevailing oxygen potential buffered by the FeO–SiO_2_ (fayalite) slag system. Collectively, these results support that Liquid#1 behaves as an interacting oxide–sulfide melt rather than a homogeneous single-component liquid.

**Figure 10:**
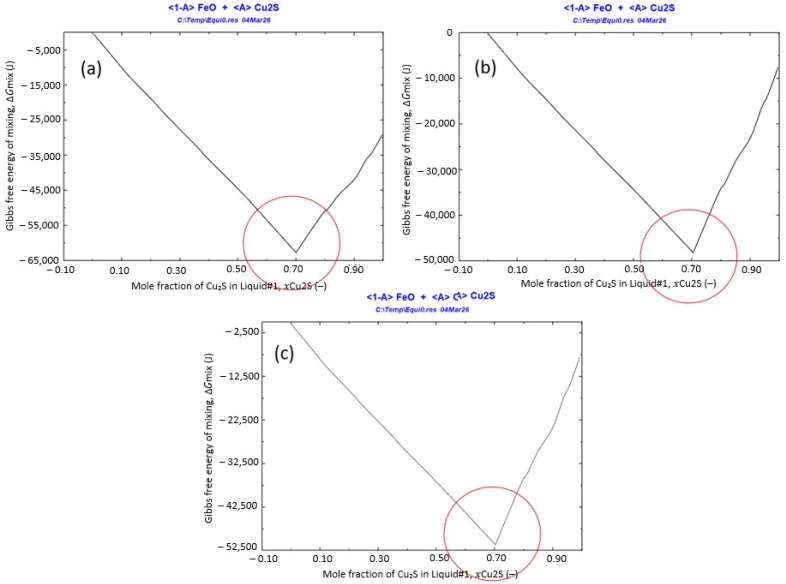
Gibbs free energy of mixing, ΔG_{mix}, of the pseudo-binary FeO–Cu_2_S liquid (Liquid#1) as a function of composition, calculated using FactSage 8.2 at (**a**) 1400 °C, (**b**) 1500 °C, and (**c**) 1600 °C. The composition is expressed as the mole fraction of Cu_2_S, x{Cu_2_S} (–), where x{FeO} = 1 − x{Cu_2_S}. The minima indicate the most thermodynamically stable compositions within the evaluated range.

To further quantify composition-dependent stability, Liquid#1 was simplified as a pseudo-binary FeO–Cu_2_S liquid, and the Gibbs free energy was calculated as a function of composition. The total amount of the pseudo-binary liquid was normalized to unity, and the composition variable was defined as the mole fraction of Cu_2_S, x(Cu_2_S), with x(FeO) = 1 − x(Cu_2_S). Figure 10a–c show the calculated Gibbs free energy variation with FeO–Cu_2_S composition at 1400, 1500, and 1600 °C, respectively. In all cases, the Gibbs free energy exhibits a clear minimum at approximately FeO:Cu_2_S ≈ 0.3:0.7 (mol/mol), indicating that a Cu_2_S-rich mixed melt is thermodynamically the most stable in this range. Notably, the position of the minimum remains nearly unchanged with temperature, suggesting weak temperature dependence of the preferred stabilization composition over 1400–1600 °C. Based on the activity trends and free-energy minimization, fayalite slag addition was subsequently designed to provide an FeO source that promotes stabilization of Liquid#1 while enabling efficient phase separation.

#### 3.3.3. Determination of Fayalite Slag Addition and Final Experimental Conditions

The final experimental conditions for the sulfidation–slag refining were established based on the thermodynamic equilibrium and stability analyses described above. The equilibrium calculations suggest that, under the present inputs (Fe–P–Cu alloy + FeS + fayalite slag), Liquid#1 is stabilized as an oxide–sulfide liquid in which Cu_2_S coexists with an FeO-bearing component. Notably, in Cu–Fe–S systems, a conventional matte-type liquid (Cu_2_S–FeS-rich) may form under sufficiently low oxygen potential; however, in our system, oxygen is buffered by the FeO–SiO_2_ slag, and thus an FeO-bearing liquid component can persist at high temperature while Cu–Fe–S intermediate sulfides may crystallize during cooling. The Gibbs free energy analysis (Section 3.3.2) identified a stable composition region near FeO:Cu_2_S ≈ 0.3:0.7 (mol/mol). Accordingly, the fayalite slag addition was determined to supply FeO to the system in a manner consistent with the free-energy-minimized stabilization of the oxide–sulfide melt, while maintaining a slag composition compatible with fayalite-based fluidity.

Reaction temperature and FeS dosage were selected as the primary experimental variables. Three temperatures (1400, 1500, and 1600 °C) were chosen to satisfy (i) complete melting of the Fe–P–Cu alloy and (ii) sufficient thermodynamic driving force for Cu sulfidation. The FeS addition was controlled stoichiometrically on a molar basis to assess the progressive effect of sulfide availability on Cu removal, using Cu:FeS molar ratios of 2:1, 2:1.5, and 2:2. For all experiments, a constant charge of 250 g Fe–7.57P–6.44Cu (wt.%) alloy was used. The sulfidation stage was conducted for 3 h after FeS addition, followed by fayalite slag addition and an additional 3 h of refining to promote phase separation. A total of nine experimental conditions were thus designed (three temperatures × three Cu:FeS ratios), as summarized in Table 6.

### 3.4. Experimental Results of Sulfidation–Slag Refining

#### 3.4.1. Microstructural and Compositional Analysis of the Recovered Fe–P Metal

**Figure 11:**
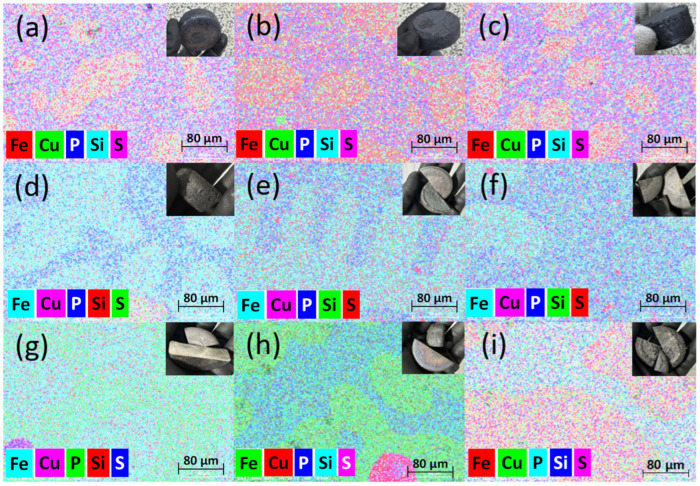
SEM–EDS elemental mapping images of recovered metal obtained under different sulfidation conditions: (**a**–**c**) 1400 °C, (**d**–**f**) 1500 °C, and (**g**–**i**) 1600 °C. For each temperature, the Cu:FeS molar ratios were (**a**,**d**,**g**) 2:1, (**b**,**e**,**h**) 2:1.5, and (**c**,**f**,**i**) 2:2.

Sulfidation–slag refining experiments were performed under the designed combinations of temperature and FeS addition, and the recovered metallic phase was examined by SEM–EDS to evaluate microstructural features and residual impurity levels. Figure 11 presents elemental mapping images of the recovered metal obtained at 1400–1600 °C with Cu:FeS molar ratios of 2:1, 2:1.5, and 2:2. Across all conditions, the recovered metal is Fe-rich, and the residual Cu is distributed relatively uniformly without forming an isolated Cu-rich metallic phase. This observation is consistent with the preferential transfer of Cu from the alloy into a sulfide-containing liquid during the sulfidation stage.

The quantitative compositions of the recovered metals are summarized in Table 7. The most favorable result was achieved at 1400 °C with Cu:FeS = 2:1, where the recovered metal exhibited the highest Fe content (90.80 wt.%) and the lowest residual Cu (1.47 wt.%). At the same temperature, increasing FeS addition led to higher residual Cu in the metal (2.77–2.97 wt.%). This trend suggests that excessive FeS does not necessarily enhance net Cu removal, but may instead promote formation and stabilization of the Cu_2_S–FeO mixed liquid (Liquid#1), increasing the likelihood of Cu repartitioning or re-dissolution between coexisting molten phases. Temperature also affected the refining outcome. At 1500–1600 °C, the recovered metals generally showed lower Fe purity and higher residual Cu. Although Cu sulfidation remains thermodynamically favorable at elevated temperatures, higher temperatures can stabilize the molten oxide–sulfide liquid and facilitate redistribution of Cu within the liquid phases, which may hinder complete separation from the metal. Therefore, the optimal condition identified in this study (1400 °C, Cu:FeS = 2:1) appears to represent a balanced regime where the sulfidation driving force and liquid-phase separation behavior jointly favor effective Cu removal and Fe(P) metal purification. Notably, phosphorus was largely retained in the Fe-rich metal phase during sulfidation–slag refining, as evidenced by the consistently high P content measured in the recovered Fe(P) metal (6.84–7.55 wt.% across all tested conditions; Table 7). Therefore, the present sulfidation–slag step primarily targets Cu partitioning, while effective P control is expected to require a subsequent dedicated dephosphorization step (e.g., controlled oxidation/slagging) after Cu removal.

#### 3.4.2. XRD Analysis of Slag–Liquid and Liquid Phases

**Figure 12:**
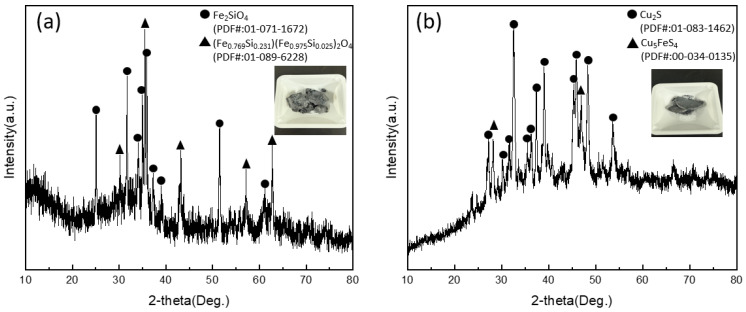
XRD patterns of (**a**) the Slag–Liquid#1 phase and (**b**) the Liquid#1 phase obtained under the optimal condition (1400 °C, Cu:FeS = 2:1). The slag-derived fraction is dominated by fayalite-related Fe–silicate phases, whereas the sulfide-rich fraction crystallized mainly as Cu_2_S with Cu–Fe–S intermediate sulfides (e.g., Cu_5_FeS_4_) after furnace cooling. The FeO-bearing component predicted for the high-temperature liquid constitution is not observed as a distinct crystalline FeO phase in XRD, likely due to incorporation into the oxide matrix and/or limited crystallinity during cooling.

Figure 12 shows the XRD patterns of (a) the Slag–Liquid#1 phase and (b) the Liquid#1 phase recovered under the optimal condition (1400 °C, Cu:FeS = 2:1). In Figure 12a, the dominant crystalline phase is fayalite (Fe_2_SiO_4_), accompanied by minor non-stoichiometric Fe–silicate solid-solution phases. These phases are plausibly formed by crystallization of a multicomponent FeO–SiO_2_-based melt during cooling, and partial Fe/Si substitution within the fayalite structure can account for the observed solid-solution reflections. Overall, the slag-side XRD confirms that a fayalite-based slag phase was stably established during refining.

In Figure 12b, Cu_2_S is identified as the major sulfide phase in the recovered Liquid#1 fraction, and Cu_5_FeS_4_ is also detected. Distinct FeO peaks are not observed, which does not necessarily contradict the presence of an FeO-bearing liquid at high temperature predicted by thermodynamic calculations. During furnace cooling, FeO can be (i) incorporated into the FeO–SiO_2_ oxide matrix and/or iron–silicate solid solutions, (ii) retained as a poorly crystalline or finely dispersed component below the XRD detection limit, and/or (iii) partially react with sulfide constituents, leading to the crystallization of Cu–Fe–S intermediate sulfides (e.g., Cu_5_FeS_4_) together with Cu_2_S. Therefore, the XRD results are consistent with a multicomponent oxide–sulfide liquid constitution at high temperature (Cu_2_S with an FeO-bearing component), while the final room-temperature crystalline assemblage after cooling is dominated by Cu_2_S and Cu–Fe–S-type sulfides. These observations support that Cu was transferred from the Fe(P) metal into a sulfide-rich liquid during refining and subsequently solidified as Cu_2_S- and Cu–Fe–S-type sulfides, consistent with the multi-component nature of Liquid#1 inferred from the thermodynamic analyses.

#### 3.4.3. Recovery Yield of Fe and Removal Efficiency of Cu

Because mass-balance evaluation requires the recovered metal mass ($M_{P}$) after phase separation, quantitative recovery/removal calculations are reported for the optimal condition for which $M_{P}$ was recorded. To quantitatively evaluate the refining performance, the Fe recovery yield and Cu removal efficiency were calculated for the optimal condition (1400 °C, Cu:FeS = 2:1). The Fe recovery yield was defined as the fraction of Fe recovered in the metallic product relative to the total Fe initially present in the feed alloy. The mass of recovered Fe was obtained by multiplying the recovered metal mass by its Fe content (wt.%) measured by SEM–EDS, and this value was normalized by the total Fe in the initial alloy:

(4) $$Fe\;recovery\;yield\;\left(\%\right)=\left(\frac{M_{P}\;\times C_{P,Fe}}{M_{F}\;\times \;C_{F,Fe}}\right)\times 100$$

where *M_P_* is the mass of recovered metal (g), *C_P_*_,*Fe*_ is the Fe content of the recovered metal (wt.%), M_F_ is the mass of the initial feed alloy (g), and *C_F_*_,*Fe*_ is the Fe content of the initial feed alloy (wt.%) Under the optimal condition, M_P_ = 206.72 g and C_P,Fe_ = 90.80 wt.%. The initial feed was *M_F_* = 250 g of Fe-7.57P-6.44Cu (wt.%), corresponding to *C_F_*_,*Fe*_ = 85.89 wt.%. Using Equation (4), the Fe recovery yield was calculated to be 87.42%. The Cu removal efficiency was defined based on the fraction of Cu remaining in the recovered metal relative to the total Cu initially present in the feed alloy:

(5) $$Cu\;removal\;efficiency\;\left(\%\right)=\left(1\;-\;\frac{M_{P}\;\times C_{P,Cu}}{M_{F}\;\times \;C_{F,Cu}}\right)\times 100$$

where $C_{P,Cu}$ is the Cu content of the recovered metal (wt.%) and $C_{F,Cu}$ is the Cu content of the initial feed alloy (wt.%). Under the optimal condition, the Cu content in the recovered Fe(P) metal decreased from 6.44 wt.% in the initial alloy to 1.47 wt.%, corresponding to a Cu removal efficiency of 81.13%.

From a process-design perspective, sulfur chemistry is relevant not only for Cu partitioning but also for the overall energy and emissions picture. Oxidation of sulfur-bearing species is exothermic; therefore, depending on reactor configuration and heat-recovery integration, sulfur oxidation may partially contribute to the thermal balance of the process. Sulfur losses to the off-gas should be minimized and managed using established gas-cleaning routes, where sulfur-containing species (e.g., SO_2_) can be captured and treated (e.g., conversion to sulfuric acid) when appropriate infrastructure is available. In scale-up, the quality of the sulfidizer (including potential trace impurities in industrial sulfide sources) should also be considered, because such impurities may partition among the metal, sulfide-rich, and slag phases and may influence separation behavior and downstream by-product handling.

## 4. Conclusions

In this study, an FeS-assisted sulfidation–slag refining process using fayalite slag was applied to an Fe–P–Cu alloy recovered from spent LiFePO_4_ (LFP) batteries via a stepwise pyrometallurgical route, with the aim of selectively removing Cu and upgrading the Fe-rich metal. The process was evaluated by combining thermodynamic calculations (FactSage 8.2) with high-temperature experiments conducted at 1400–1600 °C while varying the Cu:FeS molar ratio.

Thermodynamic equilibrium calculations confirmed that Cu has a stronger affinity for sulfur than Fe, enabling preferential Cu sulfidation to Cu_2_S. The calculations further indicated that, within the investigated compositional window in the presence of fayalite slag, a stable mixed liquid region (Liquid#1) can form in which Cu_2_S coexists with an FeO-bearing component. Moreover, the stability and amount of Liquid#1 increased with increasing Cu_2_S generation, identifying Cu_2_S formation as a key factor governing liquid-phase stabilization and, consequently, the extent of phase separation between the metallic phase and sulfide/oxide liquids.

Experimental characterization by SEM–EDS and XRD demonstrated that the recovered metal after sulfidation–slag refining was Fe-rich, whereas Cu predominantly partitioned into sulfide-associated phases and/or phases linked to the slag. The refining performance depended strongly on temperature and FeS dosage. The most favorable result was obtained at 1400 °C with Cu:FeS = 2:1, yielding the highest Fe content (90.80 wt.%) and the lowest residual Cu (1.47 wt.%) in the recovered metal. In contrast, excessive FeS addition and/or higher temperature tended to stabilize the Cu_2_S–FeO mixed liquid, thereby promoting Cu redistribution (or partial re-dissolution) between coexisting molten phases and reducing separation efficiency.

Under the optimal condition (1400 °C, Cu:FeS = 2:1), quantitative evaluation based on the recorded recovered metal mass and SEM–EDS compositions gave an Fe recovery yield of 87.42% and a Cu removal efficiency of 81.13%. In addition, phosphorus was largely retained in the Fe-rich metal phase during sulfidation–slag refining (6.84–7.55 wt.% P in the recovered Fe(P) metal across the tested conditions), indicating that the present step primarily targets Cu partitioning and that effective P control should be addressed in a subsequent dedicated dephosphorization step after Cu removal. Overall, these results demonstrate that the proposed FeS-based sulfidation–slag refining strategy enables effective and selective Cu removal from Fe-based alloys derived from spent LFP batteries and provides a process-design basis for staged upgrading of Fe-rich secondary resources.

From an economic standpoint, the proposed stepwise refining concept upgrades Fe-rich secondary resources by selectively removing Cu via a simple sulfidation-and-separation step while employing fayalite slag that can be sourced as an industrial by-product. The overall economics will depend on local FeS cost, the by-product handling strategy for the Cu-bearing sulfide phase, and the market value of the upgraded Fe product. In industrial implementation, sulfur losses and off-gas management should be considered; sulfur-bearing species can be captured and potentially valorized (e.g., conversion to sulfuric acid) using established gas-treatment routes. In addition, the quality of industrial sulfide sources (including trace impurities) may influence phase partitioning and should be accounted for in scale-up and by-product handling strategies.

## Figures and Tables

**Table 1:** Chemical composition of the Fe–P–Cu reference alloy determined by ICP–OES (wt.%).

Element	Fe	P	Cu	Al	Ca	Si	Sum.
wt.%	85.89	7.57	6.44	0.03	0.04	0.03	100

**Table 2:** Chemical composition of the fayalite-based slag determined by SEM–EDS (wt.%).

Element	Fe	Si	O	Al	C	Sum.
wt.%	62.98	16.01	14.35	3.59	3.07	100

**Table 3:** Temperature dependence of standard Gibbs free energy changes (ΔG°) for the sulfidation reactions of Fe and Cu and for the Cu–FeS exchange reaction (1400–1600 °C).

Temp. (°C)	ΔG° (2Fe + S_2_(g) → 2FeS) (kJ)	ΔG° (4Cu + S_2_(g) → 2Cu_2_S) (kJ)	ΔG° (2Cu + FeS → Fe + Cu_2_S) (kJ)
1400	−125.395	−164.958	−19.781
1500	−118.388	−158.020	−19.816
1600	−110.446	−151.096	−20.325

**Table 4:** Equilibrium compositions (mol basis) of Liquid#1 and the slag liquid phase (Slag–liq#1) calculated at different temperatures using FactSage 8.2.

Temp. (°C)	Liquid#1 (mol)				Slag–Liq#1 (mol)	
-	Cu	S	Fe	O	FeO	SiO_2_
1400	0.52782	0.26043	0.10603	0.10572	0.60427	0.38470
1500	0.52676	0.25943	0.10679	0.10702	0.60326	0.38477
1600	0.52584	0.25847	0.10745	0.10823	0.60222	0.38470

**Table 5:** Activities of elements in the Liquid#1 phase at different temperatures.

Temp. (°C)	a(Cu)	a(Fe)	a(O)	a(S)
1400	3.70 × 10^−1^	1.94 × 10^−9^	1.03 × 10^−16^	1.04 × 10^−3^
1500	3.71 × 10^−1^	2.05 × 10^−9^	2.82 × 10^−16^	1.46 × 10^−3^
1600	3.81 × 10^−1^	3.14 × 10^−9^	6.97 × 10^−16^	1.99 × 10^−3^

**Table 6:** Experimental conditions for sulfidation–slag reaction experiments.

No.	Crucible	Slag (g)	Cu (mol)	FeS (mol)	Ratio (Cu:FeS)	Temp. (°C)	FeS Time (h)	Slag Time (h)
1	Alumina	6.0128	0.2754	0.1377	2:1	1400	3	3
2		6.0128	0.2754	0.2065	2:1.5	1500	3	3
3		6.0128	0.2754	0.2754	2:2	1600	3	3
4		6.0128	0.2754	0.1377	2:1	1400	3	3
5		6.0128	0.2754	0.2065	2:1.5	1500	3	3
6		6.0128	0.2754	0.2754	2:2	1600	3	3
7		6.0128	0.2754	0.1377	2:1	1400	3	3
8		6.0128	0.2754	0.2065	2:1.5	1500	3	3
9		6.0128	0.2754	0.2754	2:2	1600	3	3

**Table 7:** SEM–EDS quantitative analysis of recovered metal under different sulfidation conditions.

No.	Temp. (°C)	Ratio Cu:FeS	Element (wt.%)				
			Fe	p	Cu	S	Si
1	1400	2:1	90.80	7.55	1.47	0.15	0.03
2	1400	2:1.5	89.40	7.36	2.77	0.40	0.07
3	1400	2:2	88.98	7.29	2.97	0.72	0.04
4	1500	2:1	89.15	7.51	3.21	0.22	0.11
5	1500	2:1.5	88.77	7.12	3.62	0.43	0.07
6	1500	2:2	88.19	7.08	3.78	0.85	0.10
7	1600	2:1	88.43	7.02	4.27	0.21	0.07
8	1600	2:1.5	87.83	7.13	4.55	0.40	0.09
9	1600	2:2	87.28	6.84	5.04	0.84	0.10

## Data Availability

The original contributions presented in this study are included in the article. Further inquiries can be directed to the corresponding author.
